# Cardiovascular complications in idiopathic pulmonary fibrosis and the emerging concept of the heart-lung fibrotic axis: a narrative review

**DOI:** 10.3389/fmed.2026.1954683

**Published:** 2026-10-14

**Authors:** Patryk Graczyk, Daria Keller, Barbara Kuźnar-Kamińska

**Affiliations:** 1 Department of Pulmonology, Allergology and Pulmonary Oncology, Poznan University of Medical Sciences, Poznań, Poland; 2 Department of Cardiology, Poznan University of Medical Sciences, Poznań, Poland

**Keywords:** antifibrotic therapy, cardiopulmonary interactions, cardiovascular disease, heart–lung axis, idiopathic pulmonary fibrosis, myocardial fibrosis, pulmonary hypertension, right ventricular dysfunction

## Abstract

Idiopathic pulmonary fibrosis (IPF) is a progressive fibrotic interstitial lung disease associated with high morbidity and mortality. Beyond respiratory failure, cardiovascular (CV) involvement is increasingly recognized as an integral component of IPF, contributing substantially to symptoms, functional decline, hospitalization, and survival. While pulmonary hypertension and right ventricular dysfunction remain the predominant CV manifestations, growing evidence suggests that subclinical myocardial dysfunction may precede overt CV disease. Collectively, these findings are consistent with a continuum of cardiopulmonary involvement, ranging from established CV complications to emerging subclinical myocardial abnormalities, rather than supporting a single unified fibrotic process. Shared profibrotic mechanisms, including transforming growth factor-β signaling, extracellular matrix remodeling, inflammation, oxidative stress, endothelial dysfunction, and neurohormonal activation, provide a biological rationale for the proposed heart–lung fibrotic axis, although its clinical relevance remains to be fully established. This review summarizes current evidence on clinical and subclinical CV manifestations in IPF, advances in multimodal diagnostic assessment, shared molecular mechanisms, and evolving therapeutic strategies. An integrated cardiopulmonary perspective may provide a framework for future studies aimed at improving risk stratification, earlier recognition of CV involvement, and development of therapeutic strategies.

## 1 Introduction

Idiopathic pulmonary fibrosis (IPF) is a chronic, progressive interstitial lung disease (ILD) characterized by the histopathological pattern of usual interstitial pneumonia and an unfavorable prognosis despite recent therapeutic advances. The disease leads to progressive fibrosis of the lung parenchyma, decline in pulmonary function, and a substantial risk of premature mortality. Its clinical course is highly heterogeneous, ranging from relatively slow functional deterioration to rapidly progressive disease and acute exacerbations (1). The clinical burden of IPF cannot be explained solely by progressive respiratory failure. Epidemiological studies consistently demonstrate a higher prevalence of cardiovascular (CV) disease in patients with IPF, including coronary artery disease (CAD), heart failure (HF), and cardiac arrhythmias, compared with age-matched general populations. A recent systematic review and meta-analysis confirmed that CV comorbidities are among the most frequent non-pulmonary conditions in this population (2).

Importantly, CV disease in IPF is not only an epidemiological association but also has clear prognostic relevance. Registry analyses have demonstrated that comorbid conditions, particularly CAD and HF, are associated with increased mortality and higher hospitalization rates, even after adjustment for lung function parameters and age. These findings suggest that CV involvement may act as an independent modifier of disease trajectory in IPF (3).

Among CV complications, pulmonary hypertension (PH) associated with interstitial lung disease (PH-ILD) plays a particularly important role. The coexistence of PH in IPF has been consistently associated with reduced exercise capacity, increased healthcare utilization, and worse survival outcomes compared with patients without PH (4, 5).

Another clinical challenge is the frequent underrecognition of CV involvement in IPF. Symptoms such as exertional dyspnea, fatigue, and reduced exercise tolerance are often attributed solely to the progression of pulmonary fibrosis, which may delay identification of concomitant cardiac abnormalities. Simultaneously, data from imaging studies indicate that subclinical cardiac dysfunction may already be present in earlier stages of disease and can be detected using advanced techniques such as myocardial deformation imaging, including global longitudinal strain (GLS) of both ventricles (6).

In parallel, growing attention has been directed toward shared molecular mechanisms of fibrosis affecting multiple organs, including activation of profibrotic signaling pathways, dysregulation of extracellular matrix (ECM) homeostasis, and chronic neurohormonal activation. Although the concept of a heart–lung fibrotic axis remains largely hypothetical, it provides a useful framework for understanding the coexistence of CV complications in patients with IPF. Against this background, the aim of this narrative review is to provide a comprehensive overview of CV complications in IPF, ranging from classical clinical entities to emerging evidence of subclinical cardiac involvement. In addition, current diagnostic approaches and therapeutic implications are discussed, with particular emphasis on their impact on prognosis and clinical management.

In this context, IPF may be better conceptualized as a lung-dominant fibrotic disorder with clinically important cardiopulmonary interactions, rather than as an exclusively pulmonary disease.

## 2 Methods

This narrative review was based on a non-systematic search of PubMed/MEDLINE, supplemented by major international society guidelines and other relevant sources. The literature search included publications available up to September 2026, with no restriction on publication date. The search focused on PH-ILD, right ventricular (RV) dysfunction, CAD, HF, arrhythmias, venous thromboembolism (VTE), myocardial deformation, cardiopulmonary exercise testing (CPET), biomarkers, antifibrotic therapies, myocardial fibrosis, shared profibrotic pathways, and cardiopulmonary interactions in IPF. Search terms included combinations of “idiopathic pulmonary fibrosis” with terms related to these clinical, diagnostic, mechanistic, and therapeutic domains. Priority was given to international guidelines, systematic reviews and meta-analyses, randomized controlled trials, and clinically relevant observational and translational studies published in English. Experimental and mechanistic studies were included when directly relevant to the proposed biological framework. Publications were selected on the basis of their relevance to the predefined clinical, diagnostic, mechanistic, and therapeutic themes of the review, with priority given to higher-level clinical evidence and landmark mechanistic studies. No formal systematic screening or evidence-grading procedure was undertaken because of the narrative design.

## 3 Classical CV complications in IPF—clinical relevance

### 3.1 PH-ILD and hemodynamic consequences

PH-ILD represents one of the most clinically important CV complications in patients with IPF. In the current classification of PH, PH resulting from chronic lung diseases and hypoxia is categorized as group 3, reflecting the close interplay among structural lung damage, pulmonary vascular remodeling, and impaired pulmonary hemodynamics. According to the most recent international guidelines, PH is defined as a mean pulmonary arterial pressure (mPAP) > 20 mmHg at rest, measured during right heart catheterization (RHC), in the presence of elevated pulmonary vascular resistance (PVR). In patients with ILDs, including IPF, this typically corresponds to a pre-capillary hemodynamic profile, defined by pulmonary arterial wedge pressure (PAWP) ≤ 15 mmHg and PVR > 2 Wood units. This revised definition facilitates earlier detection of pulmonary vascular involvement in IPF, where PH reflects vascular remodeling superimposed on progressive parenchymal fibrosis (4).

The prevalence of PH in IPF varies substantially depending on disease severity and diagnostic methodology. Contemporary observational studies suggest that PH may be present in approximately 10%–20% of patients at the time of IPF diagnosis, while the prevalence increases markedly in advanced disease and may reach 30%–50% in patients with severe functional impairment or those undergoing evaluation for lung transplantation (5, 7, 8). Together, these data indicate that pulmonary vascular involvement is a frequent and progressive component of fibrotic lung disease.

The pathophysiology of PH in IPF is multifactorial, including both parenchymal and vascular mechanisms. Progressive destruction of the pulmonary vascular bed, driven by fibrotic remodeling, chronic hypoxic vasoconstriction, endothelial dysfunction, and structural changes within the pulmonary circulation, collectively contributes to increased PVR and impaired pulmonary blood flow (7, 9).

Hemodynamically, the resulting increase in PVR imposes chronic pressure overload on the RV. Over time, this process may lead to RV hypertrophy, impaired right ventricle–pulmonary artery (RV–PA) coupling, and ultimately RV dysfunction and right HF in advanced stages of the disease. Importantly, numerous clinical studies have demonstrated that the presence of PH in IPF is associated with reduced exercise capacity, greater symptom burden, and significantly worse survival outcomes compared with patients without pulmonary vascular involvement (8, 9).

Despite its prognostic importance, early PH-ILD remains difficult to recognize because its clinical manifestations often overlap with those of progressive fibrotic lung disease. In practice, suspected pulmonary vascular involvement should prompt a structured evaluation with echocardiography and, when clinically indicated, confirmatory RHC (4).

From a therapeutic perspective, management of PH-ILD has historically been limited, with most treatment strategies focused on optimization of the underlying lung disease and supportive care. However, inhaled treprostinil has emerged as the first approved pharmacological therapy specifically for PH-ILD, demonstrating improvement in exercise capacity and functional status in clinical trials. These findings highlight that, although treatment options remain limited, pulmonary vascular involvement in IPF is not exclusively managed with supportive strategies and may represent a modifiable component of disease burden (10).

RV dysfunction represents a critical downstream consequence of pulmonary vascular involvement in IPF and constitutes a key determinant of clinical deterioration once PH develops. In patients with IPF, progressive fibrotic destruction of the pulmonary vascular bed, together with hypoxia-mediated vasoconstriction and endothelial dysfunction, results in a gradual increase in PVR. The resulting rise in RV afterload initiates a compensatory phase characterized by RV hypertrophy and increased contractile effort; however, persistent pressure overload ultimately leads to maladaptive remodeling, impaired RV–PA coupling, and progressive decline in RV performance (11, 12). Overall, PH and its RV consequences are key determinants of disease severity and prognosis in IPF.

### 3.2 CAD in patients with IPF

CAD is increasingly recognized as a clinically relevant comorbidity in patients with IPF and appears to occur more frequently in this population than would be expected based on age and traditional CV risk factors alone. Observational studies have reported a higher prevalence of ischemic heart disease among patients with IPF compared with other ILD. For example, in a cohort of patients with advanced IPF evaluated for lung transplantation, Nathan et al. reported a prevalence of angiographically confirmed CAD of approximately 60%, including a substantial proportion of previously unrecognized significant coronary stenosis, suggesting that coronary atherosclerosis may represent an important component of the CV burden associated with fibrotic lung disease (13, 14).

Analyses based on large population-based studies further support this association. In a nationwide hospital care study, patients hospitalized with IPF demonstrated a higher prevalence of conventional CV risk factors, including hypertension, diabetes, dyslipidemia, and tobacco exposure, and IPF itself remained independently associated with increased risk of CAD after multivariable adjustment (15). These findings suggest that the relationship between IPF and CAD may extend beyond shared demographic characteristics and involve systemic mechanisms.

Imaging studies provide additional evidence supporting this association. Using computed tomography to quantify coronary artery calcium burden, Bray et al. demonstrated that patients with IPF exhibited significantly greater coronary calcification compared with both patients with chronic obstructive pulmonary disease and controls who had never smoked, even after adjustment for major CV risk factors (16). These findings raise the possibility that vascular injury and accelerated atherosclerosis may occur in parallel with pulmonary fibrosis.

The mechanisms linking IPF and CAD are likely multifactorial. In addition to shared CV risk factors such as aging and smoking, chronic systemic inflammation, endothelial dysfunction, oxidative stress, and vascular injury mediated by immune mechanisms have been proposed as contributors to accelerated atherosclerosis in fibrotic lung disease (15, 16). Clinically, concomitant CAD may remain underrecognized because symptoms are often nonspecific in patients whose functional limitation is already attributed to fibrotic lung disease, potentially delaying recognition of clinically relevant myocardial ischemia (17).

### 3.3 HF in patients with IPF

HF represents an important CV comorbidity in patients with IPF, affecting approximately 10%–20% of patients depending on disease stage and study population (13, 18). Its development is multifactorial, driven by chronic hypoxemia, pulmonary vascular remodeling, ventricular interdependence, systemic inflammation, endothelial dysfunction, and neurohormonal activation, which collectively promote myocardial remodeling and diastolic dysfunction, predominantly resulting in heart failure with preserved ejection fraction (HFpEF) (19, 20). Clinically, distinguishing HF from progressive pulmonary disease remains challenging because both conditions share similar symptoms, potentially delaying recognition of cardiac involvement. Echocardiography remains the primary imaging modality for evaluating cardiac structure and function, with assessment of left ventricular (LV) systolic function, diastolic parameters, and filling pressures facilitating the identification of coexisting HF. Circulating biomarkers, particularly natriuretic peptides, provide complementary information regarding cardiac stress, although their interpretation may be confounded by concomitant PH and RV dysfunction (21, 22). Collectively, these findings indicate that HF should be regarded as an integral component of the clinical spectrum of IPF rather than merely a coincidental comorbidity, contributing substantially to symptom burden, hospitalization risk, and overall disease trajectory (22). An overview of the major CV complications in IPF, including their proposed mechanisms, diagnostic approaches, and clinical relevance, is summarized in Table 1.

**Table 1:** Cardiovascular involvement in idiopathic pulmonary fibrosis.

Cardiovascular manifestation	Main mechanisms	Key diagnostic tools	Clinical relevance
PH-ILD	Hypoxia, loss of pulmonary vascular bed, endothelial dysfunction, and pulmonary vascular remodeling (7, 9)	Echocardiography; RHC for hemodynamic confirmation; CPET for functional assessment (4, 59)	Reduced exercise capacity and adverse prognosis (4, 5)
RV dysfunction and impaired RV–PA coupling	Increased RV afterload, maladaptive RV remodeling, and impaired RV–PA coupling (11, 12)	TAPSE, FAC, RV strain, and TAPSE/PASP (12, 35, 36, 38)	RV dysfunction may occur before advanced hemodynamic disease and is associated with adverse prognosis (11, 35, 37)
CAD	Conventional CV risk factors together with systemic inflammation, oxidative stress, endothelial dysfunction, and vascular injury (15, 17)	Coronary artery calcium assessment on CT; coronary angiography when clinically indicated (14, 16)	May remain clinically underrecognized and contributes to adverse CV burden and prognosis (13, 14)
Cardiac arrhythmias	Atrial stretch, hypoxemia, pulmonary hypertension related right heart remodeling, and electrical instability (23, 24)	ECG and ambulatory ECG monitoring (23, 24)	May contribute to clinical deterioration and HF decompensation, particularly in patients with pulmonary vascular disease (25, 27)
VTE/PE	Endothelial injury, inflammation, and activation of coagulation pathways (28, 29)	CTPA in patients with suspected PE (30, 31)	Important differential diagnosis in acute respiratory deterioration; management differs substantially from acute IPF exacerbation (30, 31)
HF	Hypoxemia, ventricular interdependence, systemic inflammation, neurohormonal activation, and diastolic dysfunction (13, 18, 19, 22)	Echocardiography and natriuretic peptides (21, 22)	Contributes to dyspnea, hospitalization risk, and adverse disease trajectory (18, 22)
Subclinical LV dysfunction	Chronic hypoxemia, pulmonary vascular disease, ventricular interdependence, systemic inflammation, and endothelial dysfunction (20)	GLS and conventional diastolic echocardiographic indices (32–34)	Cardiac functional abnormalities may be detectable at or before overt CV disease, although their IPF specific and prognostic significance remains incompletely defined (32–34)
Potential myocardial fibrotic remodeling	TGF-β signaling, fibroblast activation, extracellular matrix remodeling, and potentially overlapping profibrotic pathways (39, 40, 53, 56)	CMR T1 mapping and ECV; FAPI-PET as an investigational molecular imaging approach (63, 65, 66)	Preliminary human imaging evidence in fibrotic lung disease suggests myocardial structural abnormalities, but coordinated cardiopulmonary fibrosis in human IPF remains unvalidated (40, 63)

CAD, coronary artery disease; CMR, cardiac magnetic resonance; CPET, cardiopulmonary exercise testing; CT, computed tomography; CTPA, computed tomography pulmonary angiography; CV, cardiovascular; ECG, electrocardiography; ECV, extracellular volume; FAC, fractional area change; FAPI-PET, fibroblast activation protein inhibitor positron emission tomography; GLS, global longitudinal strain; HF, heart failure; IPF, idiopathic pulmonary fibrosis; LV, left ventricular; PASP, pulmonary artery systolic pressure; PE, pulmonary embolism; PH-ILD, pulmonary hypertension associated with interstitial lung disease; RHC, right heart catheterization; RV, right ventricular; RV–PA, right ventricular–pulmonary artery; TAPSE, tricuspid annular plane systolic excursion; TGF-β, transforming growth factor-β; VTE, venous thromboembolism.

### 3.4 Cardiac arrhythmias

Cardiac rhythm disturbances are another clinically relevant CV complication in patients with IPF. Progressive pulmonary vascular remodeling and the development of PH impose chronic pressure overload on the right heart, which may contribute to atrial structural and electrical remodeling, creating a substrate for supraventricular arrhythmias (23, 24).

Available evidence indicates that atrial arrhythmias, particularly atrial fibrillation and atrial flutter, represent the most reported rhythm disturbances in patients with IPF (23). Observational studies and clinical reviews suggest that arrhythmias may occur more frequently in patients with fibrotic lung disease than in the general population. For example, in a cohort of patients with PH, Tongers et al. reported a cumulative incidence of supraventricular arrhythmias of approximately 11.7%, with an annual incidence of 2.8%, highlighting the clinical relevance of arrhythmias in this population (25). However, available data remain limited and are largely derived from observational studies (23, 26).

The mechanisms linking IPF with arrhythmogenesis are likely multifactorial and have been proposed based on pathophysiological and observational data. Chronic hypoxemia, pulmonary vascular remodeling, and increased PVR may result in elevated right atrial pressure and atrial dilation. Persistent atrial stretch and structural remodeling may promote atrial fibrosis and electrical instability, facilitating both focal and re-entrant arrhythmogenic activity (24). In addition, systemic processes associated with IPF, including chronic inflammation, oxidative stress, and endothelial dysfunction, may contribute to myocardial remodeling and electrophysiological alterations within the atrial myocardium.

From a hemodynamic perspective, rhythm disturbances may have important clinical consequences in patients with fibrotic lung disease who already have limited cardiopulmonary reserve. Evidence from PH populations suggests that supraventricular arrhythmias may impair cardiac output through loss of atrial contraction and atrioventricular dyssynchrony, which may lead to clinical deterioration in patients with RV dysfunction (27). In this setting, the onset of atrial arrhythmias has been associated with clinical deterioration, including worsening right HF, increased hospitalization rates, and poorer clinical outcomes (25, 27).

In clinical practice, arrhythmias in IPF are likely underrecognized, particularly when worsening exercise tolerance or dyspnea is attributed exclusively to pulmonary disease progression. This is clinically relevant because loss of atrial contribution to ventricular filling and impaired atrioventricular synchrony may be poorly tolerated in patients with limited cardiopulmonary reserve (23).

### 3.5 VTE and pulmonary embolism in IPF

VTE, including pulmonary embolism (PE), has emerged as an important vascular complication in patients with IPF. Population studies have demonstrated that individuals with IPF exhibit a significantly higher risk of venous thromboembolic events compared with the general population. This increased risk appears to persist even after adjustment for conventional thrombotic risk factors such as age, smoking history, and comorbid CV disease, suggesting that pathophysiological mechanisms may contribute to an increased thrombotic risk in fibrotic lung disorders (28).

Several pathophysiological mechanisms may explain the association between IPF and thromboembolism. Fibrotic lung diseases are characterized by chronic inflammation and endothelial injury, and experimental studies suggest activation of coagulation pathways within the pulmonary microenvironment. Experimental and translational studies have demonstrated that procoagulant mediators, including tissue factor and thrombin, may be upregulated in fibrotic lung tissue, linking activation of coagulation cascades with the progression of pulmonary fibrosis. These findings support the hypothesis that thrombosis and fibrosis may share overlapping biological pathways (29).

From a clinical perspective, PE may be particularly difficult to recognize in patients with IPF. Acute PE frequently presents with abrupt worsening of dyspnea, hypoxemia, and reduced exercise tolerance, symptoms that closely resemble those observed during acute exacerbations of IPF. Because these events represent a widely recognized cause of sudden respiratory deterioration in these patients, PE may occasionally be overlooked in the differential diagnosis (30).

Radiological evaluation plays a crucial role in distinguishing these entities. Computed tomography pulmonary angiography (CTPA) remains the standard diagnostic method for suspected PE, whereas acute exacerbation of IPF typically presents with new bilateral ground-glass opacities or diffuse alveolar damage superimposed on preexisting fibrotic changes on high-resolution computed tomography. Accurate differentiation is essential because the therapeutic strategies differ substantially: PE requires prompt anticoagulation therapy, whereas acute exacerbation of IPF is usually managed with supportive and anti-inflammatory approaches (30, 31).

## 4 Subclinical cardiac involvement in patients with IPF—an emerging dimension of CV complications

CV involvement in IPF extends beyond overt clinical entities such as PH, CAD, or HF. Subclinical cardiac abnormalities may develop early in the course of disease and remain undetected during routine cardiopulmonary evaluation. Advanced imaging techniques, particularly myocardial deformation analysis, have revealed subtle abnormalities in ventricular mechanics despite preserved conventional measures of cardiac function (6).

These findings highlight the complex interaction between the pulmonary and CV systems in fibrotic lung disease. Progressive pulmonary vascular remodeling, chronic hypoxemia, systemic inflammation, and endothelial dysfunction may collectively influence myocardial structure and function. Consequently, cardiac involvement in IPF is increasingly considered a continuum ranging from subclinical myocardial impairment to overt CV disease, rather than a late complication confined to advanced PH (20, 32).

### 4.1 Subclinical LV dysfunction in patients with IPF

Although LV systolic function is usually preserved in patients with IPF, several studies suggest that subclinical abnormalities in LV diastolic function may occur even in the absence of overt HF. The mechanisms underlying these alterations are likely multifactorial. They may include chronic hypoxemia, pulmonary vascular disease, ventricular interdependence, and systemic processes such as inflammation and endothelial dysfunction associated with fibrotic lung disease (20).

Echocardiographic studies have demonstrated that patients with IPF may exhibit changes in parameters reflecting impaired LV relaxation and increased filling pressures. Alterations in Doppler-derived indices, including the E/A ratio and E/e′ ratio, as well as structural parameters such as left atrial enlargement, have been reported even in individuals without clinically apparent CV disease. These findings suggest that early disturbances in ventricular compliance may precede overt manifestations of HF (33). More recently, Faverio et al. evaluated CV structural and functional parameters in 50 patients with IPF at the time of diagnosis, before initiation of antifibrotic therapy, and 50 age and sex matched controls. Diastolic dysfunction and HF were frequently identified in the IPF group; however, their prevalence did not differ significantly from the control group. These findings highlight the frequency of CV abnormalities already detectable at disease diagnosis, while also emphasizing the uncertainty regarding their specific relationship with IPF and the need for longitudinal studies (32).

More recently, myocardial deformation imaging has provided additional insight into subtle myocardial involvement. Studies using speckle tracking echocardiography suggest that LV GLS may be reduced in patients with fibrotic ILD, including those with IPF, despite preserved ejection fraction and otherwise normal conventional echocardiographic indices (34). From a clinical perspective, the presence of diastolic dysfunction may contribute to exercise intolerance and exertional dyspnea in patients with IPF (20, 33).

### 4.2 Early RV dysfunction in IPF and its potential prognostic significance

RV dysfunction may develop early in IPF, even before overt PH. Importantly, RV impairment may precede hemodynamically advanced disease and may be underestimated by conventional echocardiographic indices (35).

The concept of RV–PA coupling has emerged as a particularly relevant framework for understanding RV adaptation to increased pulmonary vascular load. Noninvasive surrogates of coupling, such as the ratio between tricuspid annular plane systolic excursion (TAPSE) and pulmonary artery systolic pressure (PASP), reflect the relationship between RV contractile function and afterload. Reduced TAPSE/PASP ratios have been associated with impaired cardiopulmonary performance and adverse outcomes in patients with fibrotic ILD, highlighting the prognostic importance of assessing RV function in relation to PVR rather than evaluating ventricular contractility in isolation (12, 36, 37).

Beyond conventional indices, assessment of RV mechanics based on strain has further improved the ability to detect early RV dysfunction. RV longitudinal strain has been shown to be a sensitive marker of RV systolic impairment and may provide incremental prognostic information in patients with pulmonary vascular disease and chronic lung disorders (38). These observations support the concept that CV involvement in IPF should not be viewed solely as a late complication of advanced PH but rather as a progressive cardiopulmonary interaction in which RV dysfunction may emerge early and contribute to exercise limitation and disease progression.

These observations underscore the importance of systematic assessment of RV structure and function in patients with IPF, particularly in those with suspected pulmonary vascular involvement. Integrating conventional echocardiographic parameters with indices of RV–PA coupling and advanced imaging techniques may improve early identification of patients at increased risk of clinical deterioration.

In summary, CV involvement in IPF may begin at a subclinical stage, with abnormalities in both LV and RV function detectable before overt CV disease. These findings support the presence of clinically relevant cardiopulmonary interactions in IPF but do not establish a shared fibrotic process involving both organs. Rather, they provide clinical context for investigating whether pulmonary and myocardial remodeling may, in selected patients, involve overlapping biological pathways. This hypothesis is discussed in the following section.

## 5 Shared molecular mechanisms of cardiac and pulmonary fibrosis: the emerging heart–lung fibrotic axis

Although IPF primarily affects the lung parenchyma, fibrotic remodeling in pulmonary and cardiac tissues may share common molecular and cellular mechanisms. Conserved biological pathways, including profibrotic signaling cascades, inflammatory mediators, oxidative stress responses, and ECM dysregulation, are implicated in fibrosis across different organs. This has led to the concept of a heart–lung fibrotic axis as a framework for understanding links between pulmonary fibrosis and cardiac remodeling (39, 40). The proposed molecular and cellular interactions underlying the heart–lung fibrotic axis, including shared profibrotic signaling pathways and downstream remodeling mechanisms, are summarized in Figure 1.

**Figure 1:**
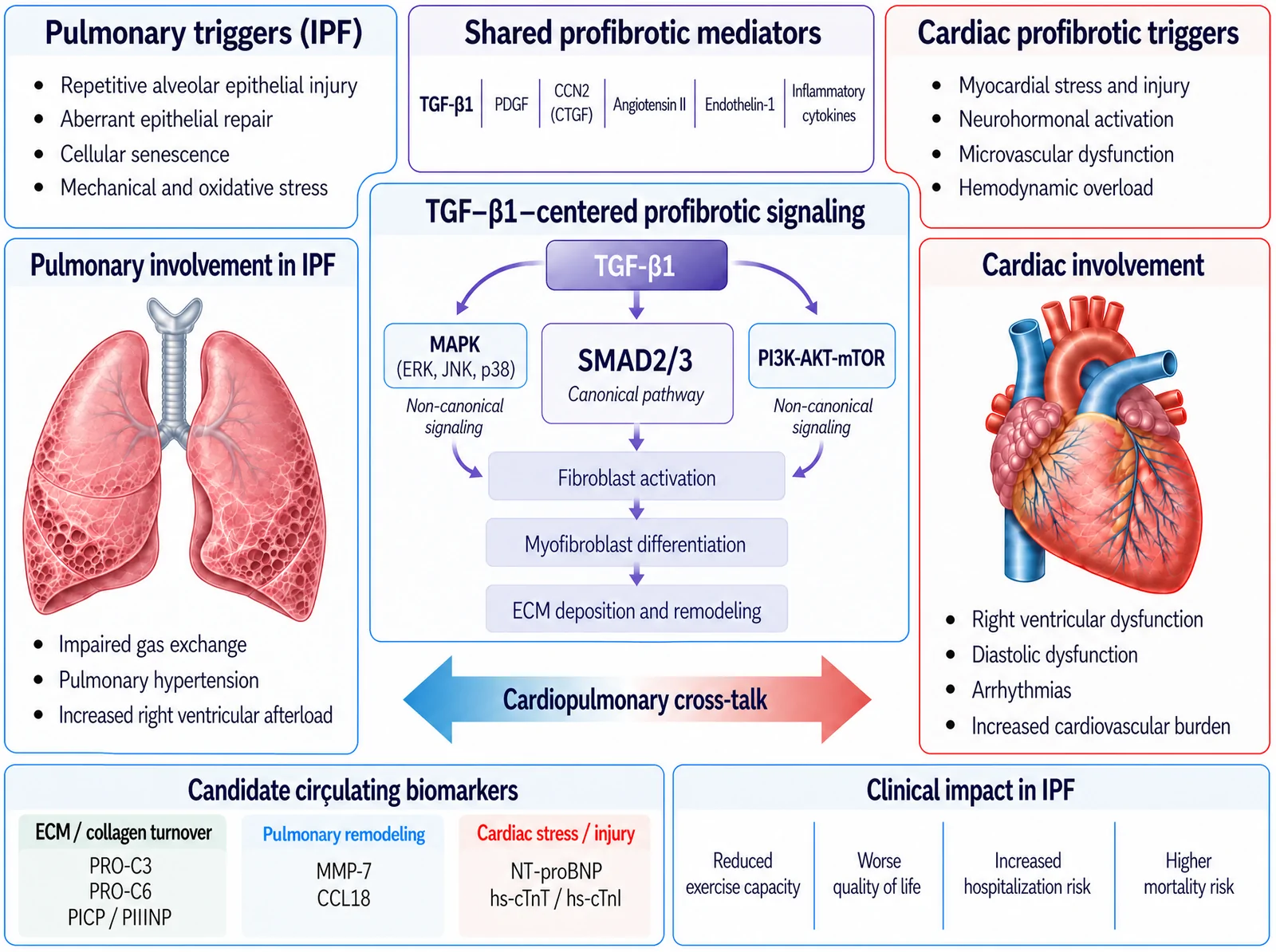
Proposed heart–lung fibrotic axis in idiopathic pulmonary fibrosis. Schematic representation of pulmonary and cardiac triggers converging on shared profibrotic mediators and TGF-β1-centered signaling pathways, with downstream fibroblast activation and extracellular matrix remodeling. The figure also summarizes potential cardiopulmonary cross-talk, associated cardiovascular and pulmonary manifestations, and candidate circulating biomarkers. The proposed molecular heart–lung axis should be regarded as a hypothesis-generating framework, as coordinated pulmonary and myocardial fibrosis has not been demonstrated prospectively in human IPF. AKT, protein kinase B; CCL18, C-C motif chemokine ligand 18; CCN2, cellular communication network factor 2 (formerly connective tissue growth factor, CTGF); ECM, extracellular matrix; ERK, extracellular signal-regulated kinase; hs-cTnI, high-sensitivity cardiac troponin I; hs-cTnT, high-sensitivity cardiac troponin T; IPF, idiopathic pulmonary fibrosis; JNK, c-Jun N-terminal kinase; MAPK, mitogen-activated protein kinase; MMP-7, matrix metalloproteinase 7; mTOR, mechanistic target of rapamycin; NT-proBNP, N-terminal pro-B-type natriuretic peptide; PDGF, platelet-derived growth factor; PI3K, phosphoinositide 3-kinase; PICP, procollagen type I C-terminal propeptide; PIIINP, procollagen type III N-terminal propeptide; PRO-C3, type III collagen formation neoepitope; PRO-C6, type VI collagen formation neoepitope; SMAD2/3, SMAD family member 2/3; TGF-β1, transforming growth factor beta 1.

Importantly, the proposed heart–lung fibrotic axis should be distinguished from established concepts such as cor pulmonale and conventional heart–lung interaction. Cor pulmonale primarily describes RV structural and functional changes that develop in response to increased pulmonary vascular load caused by chronic lung disease (41). Conventional heart–lung interaction refers more broadly to the physiological and hemodynamic interdependence between the respiratory and CV systems, including the effects of intrathoracic and alveolar pressure changes on cardiac loading conditions (42). In contrast, the heart–lung fibrotic axis proposed in this review extends beyond these established hemodynamic concepts by integrating clinically observed CV involvement in IPF with the hypothesis that pulmonary and myocardial remodeling may involve overlapping profibrotic molecular pathways. At present, this concept should be regarded as a hypothesis generating integrative framework rather than a validated disease entity, diagnostic construct, or therapeutic paradigm.

### 5.1 Central profibrotic signaling pathways and their amplification

Transforming growth factor-β (TGF-β) is a central regulator of fibroblast activation and ECM production in both pulmonary and cardiac fibrosis. TGF-β1 activates canonical signaling mediated by SMAD family members 2 and 3 (SMAD2/3), as well as noncanonical pathways, including mitogen-activated protein kinase (MAPK) signaling through extracellular signal-regulated kinase 1/2 (ERK1/2), c-Jun N-terminal kinase (JNK), and p38 MAPK, together with the phosphoinositide 3-kinase (PI3K)–protein kinase B (AKT)–mechanistic target of rapamycin (mTOR) pathway (43–45). In IPF, sustained TGF-β signaling is associated with aberrant repair following alveolar epithelial injury, whereas in the myocardium similar profibrotic pathways may be activated by mechanical stress, neurohormonal stimulation, and inflammation (39, 46, 47).

Several interacting profibrotic mediators can converge on and amplify this TGF-β-centered network. Platelet-derived growth factor (PDGF) contributes to profibrotic signaling by promoting fibroblast proliferation, migration, and activation (44). Cellular communication network factor 2 (CCN2), formerly known as connective tissue growth factor (CTGF), is another profibrotic mediator involved in fibroblast activation and ECM deposition (43). Activation of the renin–angiotensin–aldosterone system (RAAS), particularly through angiotensin II, promotes fibroblast activation, TGF-β expression, oxidative stress, and ECM accumulation (48, 49). Endothelin-1 may further promote fibroblast activation and myofibroblast differentiation through ERK1/2 signaling (43). Reactive oxygen species (ROS) generated through nicotinamide adenine dinucleotide phosphate (NADPH) oxidases (NOX) can further enhance profibrotic signaling and endothelial dysfunction (50). Inflammatory mediators also contribute to this network. Interleukin-6 (IL-6), interleukin-11 (IL-11), and C-C motif chemokine ligand 2 (CCL2) signaling have been implicated in fibroblast activation, inflammatory cell recruitment, and maintenance of a profibrotic microenvironment (47, 51, 52). Beyond the core pathways depicted in Figure 1, Janus kinase/signal transducer and activator of transcription 3 (JAK/STAT3) represents an additional inflammatory-profibrotic signaling node, particularly in cytokine-driven profibrotic responses (47). IL-6 can activate JAK/STAT3 signaling, thereby linking inflammatory signaling with fibroblast activation and profibrotic gene expression (47).

### 5.2 Fibroblast activation and ECM remodeling

These signaling networks ultimately converge on fibroblast activation and myofibroblast differentiation. Myofibroblasts may arise through several cellular routes, including activation of resident fibroblasts, mesenchymal transition processes, and recruitment of circulating fibrocytes. Once activated, they produce ECM proteins, particularly collagens I and III, and acquire contractile features that contribute to progressive tissue stiffening and architectural distortion (53–55).

ECM accumulation is further stabilized by collagen cross-linking, including lysyl oxidase-mediated processes, which increase matrix stiffness and impair tissue compliance (56). Transcriptomic and bioinformatic studies have also identified overlapping signatures related to ECM organization, inflammation, and fibroblast activation in pulmonary and cardiac fibrosis, providing additional mechanistic support for shared fibrotic biology (40, 53).

### 5.3 Evidence for cardiopulmonary interaction and current limitations

Experimental models provide proof of concept that cardiac and pulmonary remodeling may influence one another. Cardiac pressure overload models have demonstrated secondary pulmonary vascular remodeling and fibrotic changes, while microphysiological systems combining pulmonary and cardiac components support the possibility of interorgan inflammatory and profibrotic signaling (40, 57).

## 6 Diagnostic evaluation of CV complications in IPF—a practical overview

CV involvement in IPF remains underrecognized, largely due to overlap between respiratory and cardiac symptoms. Exertional dyspnea, fatigue, and reduced exercise tolerance are hallmark manifestations of IPF but may also reflect concomitant CV pathology, including PH, RV dysfunction, or HF. Identification of a CV component, therefore, requires a structured and integrative approach that combines clinical assessment, functional testing, and advanced imaging.

A CV contribution should be suspected in patients with symptoms disproportionate to pulmonary impairment, particularly when parameters such as forced vital capacity (FVC) and diffusing capacity for carbon monoxide (DLCO) do not fully account for symptom severity. Additional indicators include unexplained decline in exercise capacity, exertional desaturation inconsistent with radiological progression, and signs of right heart dysfunction such as peripheral edema or elevated jugular venous pressure (4, 58).

CPET provides an integrative assessment of ventilatory and circulatory responses and enables differentiation between ventilatory and circulatory limitation. Findings suggestive of CV involvement include reduced peak oxygen uptake (VO_2_ peak), decreased oxygen pulse (VO_2_/HR) reflecting impaired stroke volume, increased ventilatory equivalent for carbon dioxide (VE/VCO_2_ slope), and early anaerobic threshold. These abnormalities indicate circulatory limitation, including reduced cardiac output or pulmonary vascular disease, rather than isolated respiratory impairment (59).

Echocardiography is the primary imaging modality for CV evaluation in IPF; however, conventional parameters may be insufficient for detecting early myocardial involvement. Left ventricular ejection fraction (LVEF) is frequently preserved and lacks sensitivity for identifying subclinical dysfunction. In contrast, GLS derived from speckle tracking echocardiography can detect subtle abnormalities in myocardial deformation and may reveal early systolic dysfunction despite preserved LVEF. Studies in IPF populations have demonstrated impaired GLS despite normal LVEF, indicating subclinical myocardial involvement not captured by conventional echocardiographic indices (6).

Assessment of RV function is crucial given the impact of pulmonary vascular remodeling. Conventional indices such as TAPSE, RV fractional area change (FAC), and estimated RV systolic pressure (RVSP) may remain within normal limits in early disease. More sensitive parameters include longitudinal strain of the RV free wall and the TAPSE/PASP ratio, which reflect coupling between the right ventricle and pulmonary artery. Impairment of these parameters may precede overt PH and represents an early marker of maladaptive RV response to increased afterload (60, 61).

Advanced echocardiographic assessment shows that left and RV strain may be impaired even in patients with mild-to-moderate IPF and preserved conventional echocardiographic parameters. Supported by systematic reviews and meta-analyses, these findings highlight the value of myocardial deformation imaging for detecting subclinical CV involvement (6).

Atrial strain analysis, particularly left atrial reservoir strain, provides additional information on diastolic function and filling pressures and may help in identifying coexisting HF with preserved ejection fraction (62).

Despite these advances, echocardiography has important limitations. Conventional indices may remain normal until advanced stages, and Doppler estimation of pulmonary artery pressure may be unreliable in ILD due to suboptimal acoustic windows. As a result, early PH and subtle myocardial dysfunction may be underestimated (4).

When noninvasive findings are inconclusive or precise hemodynamic assessment is required, RHC remains the gold standard for PH diagnosis, enabling direct measurement of mPAP, PVR, and PAWP (4).

CTPA is essential for excluding PE and may reveal indirect signs of PH, including enlargement of the main pulmonary artery.

Cardiac magnetic resonance (CMR) has emerged as a valuable modality for assessing myocardial structure and function. Techniques such as T1 mapping and extracellular volume (ECV) quantification enable the detection of myocardial fibrosis, while precise volumetric analysis allows detailed assessment of ventricular remodeling. Prospective studies in patients with fibrotic lung diseases suggest that CMR may detect myocardial abnormalities not captured by echocardiography, supporting its role in the assessment of subclinical cardiac involvement in patients with fibrotic lung diseases, including those with IPF (63).

Emerging molecular imaging techniques targeting fibrotic activity are also gaining increasing attention. Fibroblast activation protein inhibitor positron emission tomography (FAPI-PET) enables visualization of activated fibroblasts and has promising results for assessing disease activity in pulmonary fibrosis. A recent prospective head-to-head study of 52 patients with IPF found that whole-lung [18F]FAPI-42 uptake, unlike [18F]fluorodeoxyglucose ([18F]FDG) uptake, independently predicted death or lung transplantation and showed stronger associations with disease severity, supporting its potential as a prognostic imaging biomarker (64). At present, clinical data primarily focus on pulmonary involvement; however, these approaches may, in the future, provide insight into systemic fibrotic processes and contribute to the evaluation of cardiopulmonary interactions (65, 66).

### 6.1 Circulating biomarkers of fibrotic and CV remodeling

Circulating biomarkers may complement functional and imaging assessment by providing dynamic information on ECM remodeling, fibrotic activity, and CV stress. In IPF, circulating markers of collagen turnover are among the most extensively investigated candidates. In the prospective PROFILE cohort, matrix metalloproteinase (MMP)-degraded type I collagen (C1M), type III collagen (C3M), and type VI collagen (C6M), together with MMP-degraded C-reactive protein (CRPM), were higher in patients with progressive disease, and longitudinal increases in several of these markers were associated with poorer survival (67).

Subsequent PROFILE analyses demonstrated that collagen type III and VI synthesis neoepitopes (PRO-C3 and PRO-C6) were elevated in patients with IPF compared with healthy controls and were higher in progressive than stable disease (68). These findings, together with subsequent studies of collagen turnover in IPF, support the potential value of circulating ECM biomarkers for disease phenotyping and prognostic assessment, although their reproducibility and validation remain insufficient for routine clinical implementation (69, 70).

Matrix metalloproteinase 7 (MMP-7) represents another extensively investigated blood biomarker in IPF. An individual participant data meta analysis demonstrated that higher baseline MMP-7 concentrations were independently associated with both mortality and disease progression (71). More recently, analysis of 1,280 patients from the ISABELA trials showed that higher baseline concentrations of MMP-7 and C-C motif chemokine ligand 18 (CCL18) identified patients at increased risk of mortality, while longitudinal changes in CCL18 provided additional information on disease progression (72).

Circulating markers of collagen turnover have also been investigated as indirect indicators of myocardial ECM remodeling. These include the C-terminal propeptide of procollagen type I (PICP), N-terminal propeptide of procollagen type I (PINP), N-terminal propeptide of procollagen type III (PIIINP), and carboxy-terminal telopeptide of type I collagen (CITP). Among these markers, PICP has shown the most consistent association with myocardial collagen burden, although circulating collagen products are not specific to the heart (73). Importantly, a systematic review of 89 CV studies demonstrated marked variability in reported PICP, PINP, PIIINP, and CITP concentrations across studies, including variability between studies using similar assays, which complicates the establishment of standardized reference ranges and clinical thresholds (74).

CV biomarkers provide complementary information but reflect a different component of the cardiopulmonary phenotype. B-type natriuretic peptide (BNP) and N-terminal pro-B-type natriuretic peptide (NT-proBNP) primarily reflect myocardial wall stress rather than fibrotic burden and may support evaluation for PH or HF in patients with ILD. Expert consensus identifies natriuretic peptides as useful adjunctive screening tools when PH-ILD is suspected, although RHC remains necessary for hemodynamic confirmation (75). In ILD cohorts, low NT-proBNP concentrations have shown a high negative predictive value for PH, whereas elevated NT-proBNP and cardiac troponin T have been associated with increased mortality (76). However, these markers primarily reflect hemodynamic stress or myocardial injury and should not be interpreted as specific measures of myocardial fibrosis (73). Accordingly, no circulating biomarker or biomarker panel has yet been prospectively validated as a specific measure of the proposed heart–lung fibrotic axis. Their current role should therefore be considered complementary, and prospective studies are needed to determine whether biomarker trajectories provide incremental information beyond pulmonary function, CV imaging, and hemodynamic assessment.

Taken together, evaluation of CV complications in IPF requires a multimodal approach integrating clinical assessment, circulating biomarkers, echocardiography with deformation imaging, CPET, and selected advanced imaging techniques. Such a multimodal strategy may facilitate the detection of subclinical cardiac involvement. However, its incremental value for routine risk stratification and the optimal combination and timing of individual modalities remain to be established.

## 7 Therapeutic strategies across the heart–lung fibrotic axis

Therapeutic management across the heart–lung fibrotic axis remains fundamentally asymmetric, with substantially different levels of evidence between the pulmonary and cardiac domains. Pirfenidone and nintedanib are established antifibrotic therapies for IPF, while nerandomilast has recently become an approved treatment option for IPF (1, 77–80). By contrast, no drug is currently approved specifically as an antifibrotic therapy for myocardial fibrosis. Consequently, clinical management combines disease-modifying treatment directed at pulmonary fibrosis with conventional CV therapies prescribed according to established CV indications, while several additional pathway-specific antifibrotic strategies remain under clinical or preclinical investigation. Recent phase III data have further expanded the therapeutic landscape: in TETON-2, the between-group difference in FVC change at week 52 favored inhaled treprostinil by 95.6 mL, and treatment was associated with a lower risk of clinical worsening (hazard ratio 0.71, 95% confidence interval 0.53–0.95) (81). Consistent findings were observed in the replicate TETON-1 trial, in which inhaled treprostinil was associated with a 130.1-mL between-group difference in FVC change at week 52 vs. placebo and a lower risk of clinical worsening (hazard ratio, 0.67; 95% confidence interval, 0.52–0.88) (82). Because the present review is intentionally restricted to IPF, disease-specific immunomodulatory therapies used in connective tissue disease-associated ILD, including JAK inhibitors, biological agents, and glucocorticoid-based regimens, are not discussed here. Their efficacy and CV implications should not be extrapolated to IPF. The main approved and emerging therapeutic strategies discussed in this section are summarized in Table 2.

**Table 2:** Therapeutic strategies across the heart–lung fibrotic axis in idiopathic pulmonary fibrosis.

Therapeutic strategy	Main target/mechanism	Current clinical status and key evidence	Relevance to the proposed heart–lung fibrotic axis
A. Current clinically available therapies for pulmonary fibrosis or pulmonary vascular disease			
Pirfenidone	Pleiotropic modulation of profibrotic signaling, including TGF-β related pathways, fibroblast activation, and extracellular matrix accumulation (39, 46)	Approved antifibrotic therapy for IPF. CAPACITY and ASCEND demonstrated attenuation of disease progression and FVC decline (77, 83, 84)	Pulmonary antifibrotic efficacy is established. Overlap with pathways involved in myocardial fibrosis provides translational rationale, but direct cardiovascular benefit in IPF has not been established (85, 92)
Nintedanib	Inhibition of PDGFR, FGFR, and VEGFR tyrosine kinase signaling, reducing fibroblast proliferation and matrix deposition (78)	Approved for IPF and other chronic fibrosing ILDs with a progressive phenotype. INPULSIS and INBUILD demonstrated attenuation of FVC decline (78, 87, 88)	Pulmonary antifibrotic efficacy is established; whether treatment modifies cardiovascular complications or myocardial remodeling remains unknown. Cardiovascular event rates were not increased vs. placebo in pooled trial analyses (89)
Nerandomilast	Preferential PDE4B inhibition with antifibrotic and immunomodulatory effects	Approved in the European Union for adults with IPF and PPF. Phase III FIBRONEER IPF and FIBRONEER ILD demonstrated attenuation of FVC decline (79, 80, 91)	Established benefit is currently pulmonary. Direct effects on myocardial fibrosis or cardiovascular outcomes have not been demonstrated
Inhaled treprostinil	Prostacyclin mediated pulmonary vasodilation targeting pulmonary vascular dysfunction	Demonstrated improvement in exercise capacity in PH-ILD in the INCREASE trial; approved in the United States for PH-ILD (10)	Targets pulmonary vascular involvement and thereby the hemodynamic component of cardiopulmonary disease; it has not been shown to modify shared pulmonary and myocardial fibrotic remodeling
B. Established cardiovascular therapies with indirect effects on myocardial remodeling			
Neurohormonal blockade (ACEIs, ARBs, MRAs, β blockers)	Modulation of RAAS and sympathetic signaling with attenuation of maladaptive myocardial remodeling; RAAS blockade may also reduce profibrotic signaling	Established cardiovascular therapies prescribed according to conventional disease specific indications (94, 95)	May influence myocardial remodeling pathways that overlap with pulmonary fibrotic biology, but these agents are not specific therapies for the proposed heart–lung fibrotic axis and should not be initiated solely for a putative antifibrotic effect (48, 49, 94)
C. Human translational evidence for antifibrotic treatment of myocardial fibrosis			
Pirfenidone in myocardial fibrosis (PIROUETTE)	Modulation of myocardial extracellular matrix remodeling	Phase II randomized trial in HFpEF with CMR defined myocardial fibrosis. Pirfenidone reduced myocardial ECV over 52 weeks compared with placebo; the trial was not designed to demonstrate hard cardiovascular outcome benefit (86)	Provides human proof of concept that an antifibrotic drug established in pulmonary disease can modify an imaging marker of myocardial fibrosis. It does not demonstrate treatment of a coordinated cardiopulmonary fibrotic process or establish pirfenidone as cardiovascular therapy
D. Investigational and experimental antifibrotic strategies			
TGF-β/ALK5 inhibition	Direct inhibition of central TGF-β dependent profibrotic signaling and downstream fibroblast activation	Preclinical evidence in experimental pulmonary fibrosis and cardiac pressure overload models; no approved ALK5 inhibitor for antifibrotic treatment (96, 97)	Strong mechanistic rationale in both pulmonary and cardiac fibrosis, but evidence for coordinated cardiopulmonary therapeutic effects remains preclinical
TNIK inhibition (rentosertib)	Inhibition of TNIK mediated profibrotic and proinflammatory signaling	Early clinical development. A randomized phase IIa trial provided initial human efficacy and safety data in IPF (98)	Represents a pathway directed pulmonary antifibrotic strategy; effects on myocardial fibrosis or an integrated cardiopulmonary phenotype have not been established
Senotherapeutic strategies	Elimination of senescent cells or modulation of the senescence associated secretory phenotype	Early clinical studies of dasatinib plus quercetin have primarily assessed feasibility and short term tolerability; not standard care (99, 100)	Biologically relevant to age associated fibrotic remodeling, but direct evidence for cardiovascular benefit or modification of a coordinated heart– lung fibrotic process is lacking
FAP-targeted therapeutic approaches	Targeting activated fibroblast populations through FAP-directed strategies	Therapeutic applications remain preclinical or early translational. FAPI-PET is currently more developed as a molecular imaging approach than as a therapeutic platform (101)	Potential future strategy for fibroblast directed intervention across organs, but therapeutic relevance to the proposed heart–lung fibrotic axis remains speculative
Cell based approaches	Mesenchymal stromal cell and iPSC-derived strategies aimed at antifibrotic or regenerative effects	Experimental; not standard care in fibrosing ILD (102)	Potential future approach to fibrotic lung disease; evidence for integrated pulmonary and myocardial benefit is currently lacking

ACEIs, angiotensin converting enzyme inhibitors; ALK5, activin receptor like kinase 5; ARBs, angiotensin receptor blockers; CMR, cardiac magnetic resonance; ECV, extracellular volume; FAP, fibroblast activation protein; FAPI-PET, fibroblast activation protein inhibitor positron emission tomography; FGFR, fibroblast growth factor receptor; FVC, forced vital capacity; HFpEF, heart failure with preserved ejection fraction; ILD, interstitial lung disease; IPF, idiopathic pulmonary fibrosis; iPSC, induced pluripotent stem cell; MRAs, mineralocorticoid receptor antagonists; PDE4B, phosphodiesterase 4B; PDGFR, platelet derived growth factor receptor; PH-ILD, pulmonary hypertension associated with interstitial lung disease; PPF, progressive pulmonary fibrosis; RAAS, renin angiotensin aldosterone system; TGF-β, transforming growth factor-β; TNIK, Traf2 and Nck interacting kinase; VEGFR, vascular endothelial growth factor receptor.

### 7.1 Clinically approved antifibrotic therapies for pulmonary fibrosis

#### 7.1.1 Pirfenidone

Pirfenidone is an established antifibrotic therapy approved for the treatment of IPF and remains an important component of contemporary disease—modifying treatment. Its mechanism of action is pleiotropic, involving modulation of multiple profibrotic pathways, particularly those related to TGF-β, as well as suppression of fibroblast activation and ECM accumulation (39, 46). Accordingly, pirfenidone is best understood as a modulator of the inflammatory fibrotic interface that interferes with several converging drivers of aberrant tissue repair.

The clinical efficacy of pirfenidone in IPF was established in the CAPACITY and ASCEND phase III trials. In CAPACITY, pirfenidone reduced the decline in FVC, with one of the two replicate studies meeting its primary endpoint and the overall program demonstrating a consistent effect in slowing disease progression (83). In ASCEND, pirfenidone significantly reduced the proportion of patients with a ≥10% decline in predicted FVC or death at 52 weeks and improved progression-free survival (77). Taken together, these findings confirm that pirfenidone slows disease progression, although it does not reverse established fibrosis, and support its role based on the overall consistency of evidence across trials (77, 83).

From a practical perspective, treatment requires monitoring for gastrointestinal intolerance, photosensitivity or rash, fatigue, weight loss, and hepatic toxicity. Liver function tests should be obtained before treatment and monitored regularly during therapy in accordance with current product information (84). Within the heart–lung axis framework, pirfenidone is particularly important because its biological effects overlap with pathways implicated not only in pulmonary fibrosis but also in myocardial ECM remodeling. This overlap provides a mechanistic rationale for translational studies of fibrotic cardiac phenotypes and makes pirfenidone the most clinically advanced example of an antifibrotic drug approved for lung disease that is now being explored beyond the lung (39, 46, 85, 86).

#### 7.1.2 Nintedanib

Nintedanib is an intracellular tyrosine kinase inhibitor approved for the treatment of IPF that targets platelet-derived growth factor receptor (PDGFR), fibroblast growth factor receptor (FGFR), and vascular endothelial growth factor receptor (VEGFR) (1, 46, 78, 87). By inhibiting these receptor tyrosine kinase pathways, nintedanib suppresses fibroblast proliferation, migration, differentiation, and matrix deposition, thereby attenuating the structural progression of fibrotic remodeling.

Its efficacy in IPF was established in the replicate INPULSIS-1 and INPULSIS-2 trials, in which nintedanib significantly reduced the annual rate of FVC decline compared with placebo over 52 weeks (78). The magnitude of effect was clinically relevant and consistent with the slowing of disease progression.

Importantly, the antifibrotic effect of nintedanib was later extended beyond IPF in the INBUILD trial, where the drug slowed the annual rate of FVC decline in patients with chronic progressive fibrosing ILDs other than IPF (87). In INBUILD, the adjusted rate of FVC decline was markedly lower with nintedanib than with placebo, supporting the concept that nintedanib targets shared fibrotic pathways across multiple ILD phenotypes rather than being limited to classical IPF. The official product information also continues to list IPF and other chronic fibrosing ILDs with a progressive phenotype among approved indications (88).

The INBUILD findings support the activity of nintedanib across different pulmonary fibrosing ILD phenotypes, but they do not establish antifibrotic efficacy in extrapulmonary organs (87). Nintedanib therefore remains a treatment directed at pulmonary fibrotic disease. Whether slowing pulmonary fibrosis translates into a reduction in CV complications or modifies myocardial remodeling has not been established.

From a safety perspective, nintedanib requires particular attention in patients with CV comorbidities. Gastrointestinal adverse events, particularly diarrhea, and elevations in liver enzymes are well recognized. Current product information also includes precautions regarding bleeding and arterial thromboembolic events, which are clinically relevant in patients with CAD and in those receiving antiplatelet or anticoagulant therapy (88). CV risk should therefore be considered as part of individualized treatment assessment and monitoring. In a pooled *post hoc* analysis of the TOMORROW and INPULSIS trials, the incidence of major adverse CV events was similar between nintedanib and placebo in patients with both higher and lower baseline CV risk (89).

#### 7.1.3 Nerandomilast

Nerandomilast is an orally administered preferential phosphodiesterase 4B (PDE4B) inhibitor with antifibrotic and immunomodulatory properties (79, 90). Following encouraging phase II findings (90), nerandomilast was evaluated in the phase III FIBRONEER-IPF and FIBRONEER-ILD trials (79, 91). In FIBRONEER-IPF, nerandomilast resulted in a smaller decline in FVC over 52 weeks than placebo (79). In FIBRONEER-ILD, a smaller decline in FVC was also demonstrated in patients with progressive pulmonary fibrosis (PPF) (91). In July 2026, nerandomilast received European Union marketing authorization for the treatment of adults with IPF and PPF (80). The pivotal trials evaluated pulmonary rather than CV endpoints, and effects on myocardial fibrosis or CV outcomes have not been established (79, 91). The most common adverse effects include diarrhea and weight loss, and dose reduction may be required to manage treatment-related adverse effects (80).

### 7.2 CV outcomes of antifibrotic therapy

Clinical trials of antifibrotic therapy in IPF have primarily been designed around pulmonary endpoints, particularly changes in FVC, and evidence for direct CV benefit remains limited. In a pooled *post hoc* analysis of the TOMORROW and INPULSIS trials, the incidence of major adverse CV events was similar between nintedanib and placebo across subgroups defined by baseline CV risk (89). Similarly, pooled analysis of three phase III pirfenidone trials demonstrated comparable rates of major adverse cardiac events and bleeding events between pirfenidone and placebo (92). Meta analytic evidence suggests that antifibrotic therapy is associated with a reduction in all cause mortality in IPF, although this finding should not be interpreted as evidence of a CV specific survival benefit (93). Effects on HF hospitalization or progression of PH have not been prospectively established as treatment benefits of antifibrotic therapy in IPF.

### 7.3 CV therapies with indirect antifibrotic effects

#### 7.3.1 Neurohormonal blockade

Unlike pulmonary fibrosis, myocardial fibrosis is not currently treated with an approved drug specifically labeled as a direct antifibrotic therapy. Instead, antifibrotic effects in CV medicine are achieved indirectly through established neurohormonal blockade. This applies primarily to angiotensin-converting enzyme inhibitors (ACEIs), angiotensin receptor blockers (ARBs), mineralocorticoid receptor antagonists, and β-blockers (47–49, 94, 95). These drugs are not classical antifibrotic agents in the same sense as pirfenidone or nintedanib; however, they target pathways involved in adverse myocardial remodeling, oxidative stress, fibroblast activation, and ECM accumulation. The RAAS is of particular importance because it constitutes a major profibrotic pathway in both the heart and the lungs. Angiotensin II promotes fibroblast activation, myofibroblast differentiation, oxidative stress, inflammation, and collagen deposition, whereas aldosterone amplifies matrix remodeling and tissue stiffness (48, 49). Consequently, ACEIs and ARBs may attenuate fibrotic remodeling by limiting RAAS-mediated signaling, while mineralocorticoid receptor antagonists further counteract effects driven by aldosterone and pathways associated with oxidative stress (49, 94, 95). β-blockers are less directly antifibrotic at a molecular level, but by reducing chronic sympathetic activation, myocardial oxygen demand, and hemodynamic stress, they may help limit maladaptive structural remodeling over time (94).

In patients with established CV indications, guideline based neurohormonal therapy may favorably influence adverse myocardial remodeling. These agents should not, however, be regarded as specific treatments for the proposed heart–lung fibrotic axis or initiated solely for a putative antifibrotic effect. CV therapy should continue to follow established disease specific indications (94, 95).

#### 7.3.2 Pirfenidone in cardiac disease

Among currently available antifibrotic drugs, pirfenidone has generated the strongest translational interest in myocardial fibrosis. Preclinical studies and mechanistic reviews suggest that pirfenidone can reduce ECM accumulation in the myocardium, attenuate collagen deposition, and improve selected indices of adverse remodeling in experimental cardiac injury models (85). This evidence provided the rationale for testing pirfenidone in patients with HFpEF, a syndrome in which diffuse myocardial fibrosis contributes importantly to diastolic dysfunction and adverse outcomes.

The PIROUETTE trial evaluated pirfenidone in patients with HFpEF and myocardial fibrosis identified on CMR imaging. After 52 weeks, pirfenidone reduced myocardial ECV, the prespecified imaging marker of diffuse myocardial fibrosis, compared with placebo (86). However, PIROUETTE was a phase II study and was not designed to establish a hard CV outcome benefit. Thus, pirfenidone remains an antifibrotic therapy approved for lung disease, with early clinical evidence in cardiac fibrosis, rather than an established therapy in cardiology (86).

### 7.4 Emerging shared targets: heart–lung candidates under investigation

#### 7.4.1 TGF-β/ALK5 inhibition

TGF-β signaling represents a central conserved pathway in pulmonary and cardiac fibrosis. Direct inhibition of activin receptor-like kinase 5 (ALK5), the type I TGF-β receptor, has therefore been investigated as a potential antifibrotic strategy. Pharmacological ALK5 inhibition attenuated progressive fibrosis in experimental pulmonary models and reduced myocardial fibrosis and collagen cross-linking in cardiac pressure overload models. However, these findings remain preclinical, and no ALK5 inhibitor is approved for routine antifibrotic treatment (96, 97).

#### 7.4.2 TNIK inhibition

Traf2- and Nck-interacting kinase (TNIK) has emerged as a potential regulator of profibrotic and proinflammatory signaling. Rentosertib, a small molecule TNIK inhibitor, was evaluated in a randomized phase IIa trial in patients with IPF and showed encouraging early efficacy and safety findings. However, this strategy remains investigational, and effects on myocardial fibrosis or an integrated cardiopulmonary phenotype have not been established (98).

#### 7.4.3 Senotherapeutic strategies

Cellular senescence may contribute to persistent fibrotic remodeling through the senescence-associated secretory phenotype, which promotes inflammation and fibroblast activation. In IPF, early clinical studies of the senolytic combination dasatinib and quercetin have primarily assessed feasibility and short-term tolerability. Although these findings provide preliminary translational support for senotherapeutic approaches, the available studies remain small and exploratory and do not support routine clinical use (99, 100).

#### 7.4.4 FAP targeting and cell-based approaches

Fibroblast activation protein (FAP) is a marker of activated fibroblasts and represents a potential theranostic target in fibrotic disease. At present, FAP-directed imaging is substantially more advanced than therapeutic targeting, whereas proposed FAP-directed antifibrotic strategies remain preclinical or early translational (101). Cell-based approaches, including mesenchymal stromal cell strategies and induced pluripotent stem cell (iPSC)-derived platforms, are also being investigated in fibrosing ILD but remain experimental and are not part of standard care (102).

### 7.5 Practical perspective on the heart–lung axis

At present, therapeutic strategies across the heart–lung fibrotic axis remain largely organ-specific rather than integrated. Pulmonary fibrosis is treated with approved antifibrotic agents, whereas cardiac involvement is managed using conventional CV therapies directed at comorbid conditions and adverse remodeling. In clinical practice, this distinction is important because CV comorbidities are common in IPF and are associated with worse prognosis, higher symptom burden, and greater therapeutic complexity (2, 13–17, 23, 24, 26, 27). In addition, CV comorbidities often complicate antifibrotic therapy because these patients are typically older, receive multiple concomitant medications, and frequently require antiplatelet or anticoagulant therapy. These factors increase the risk of drug interactions, bleeding complications, particularly with nintedanib, and treatment-related adverse events, necessitating careful patient selection and monitoring. In practice, treatment should remain indication based and individualized according to pulmonary disease severity, CV comorbidity burden, concomitant medication use, and treatment tolerability. Liver function and gastrointestinal adverse effects require monitoring during antifibrotic therapy, while bleeding and arterial thromboembolic risk deserve particular attention in patients receiving nintedanib, especially in the presence of concomitant antiplatelet or anticoagulant treatment (88). With nerandomilast, gastrointestinal tolerability and weight loss should also be monitored (80). No currently available therapy should be prescribed specifically to modify the proposed heart–lung fibrotic axis outside its established clinical indication.

## 8 Discussion

### 8.1 Interpretation of current evidence

CV disease constitutes an important component of the clinical burden of IPF, encompassing both conventional CV comorbidities and manifestations related to pulmonary vascular and cardiopulmonary dysfunction. The current literature supports a continuum extending from pulmonary vascular disease and RV dysfunction to CAD, HF, arrhythmias, VTE, and subclinical myocardial abnormalities detectable with advanced imaging. Collectively, these findings reinforce the concept that CV involvement in IPF should be interpreted within an integrated cardiopulmonary framework rather than as isolated organ-specific manifestations. This perspective provides a coherent framework for understanding the clinical spectrum of CV complications in IPF while highlighting the potential links between pulmonary fibrosis, pulmonary vascular remodeling, cardiac dysfunction, and shared profibrotic mechanisms, which are discussed in greater detail in the following sections.

### 8.2 Limitations of current evidence and knowledge gaps

Despite growing recognition of CV involvement in IPF, several important knowledge gaps remain. First, there is still a lack of prospective studies specifically designed to detect subclinical cardiac involvement in patients with IPF at early disease stages. Most currently available data are derived from cross-sectional studies, retrospective analyses, or relatively small observational cohorts, which limits the ability to determine the temporal sequence, prevalence, and prognostic significance of these abnormalities detected by advanced echocardiography or CMR (6, 20, 33, 34, 41, 63, 103, 104). As a result, it remains uncertain whether subtle alterations in LV strain, RV mechanics, or myocardial fibrosis represent early independent manifestations of systemic fibrotic remodeling, secondary consequences of pulmonary vascular disease, or epiphenomena associated with advanced cardiopulmonary impairment.

#### 8.2.1 Evidence levels supporting the heart–lung fibrotic axis

The evidence supporting the proposed heart–lung fibrotic axis can be considered at three distinct levels. First, established clinical evidence demonstrates a substantial CV burden in IPF, including PH, RV dysfunction, CAD, HF, arrhythmias, and VTE, with several of these manifestations carrying established prognostic significance (2, 5, 12, 13, 28). Second, human imaging studies provide emerging evidence of subclinical myocardial involvement, including abnormalities in ventricular mechanics despite preserved conventional measures of cardiac function (6, 34). CMR studies in fibrotic lung disease have also provided preliminary evidence of myocardial structural abnormalities (63). Third, evidence supporting shared molecular fibrotic mechanisms between the heart and lungs remains predominantly mechanistic, translational, or experimental (40, 57). Although overlapping profibrotic pathways are well documented in pulmonary and cardiac fibrosis when studied separately (39, 40, 53, 56), direct demonstration of a coordinated cardiopulmonary fibrotic process in human IPF is currently lacking. Accordingly, the proposed heart–lung fibrotic axis should be regarded as a hypothesis-generating framework rather than a validated clinical paradigm.

#### 8.2.2 Potential clinical value and current limitations

At present, the clinical value of this framework is primarily conceptual rather than prescriptive. Its potential advantage over conventional models is that it integrates established CV complications with subclinical myocardial abnormalities and the possibility of shared fibrotic biology. In clinical practice, this framework may support earlier CV assessment when dyspnea, exercise limitation, or functional deterioration appears disproportionate to the severity of pulmonary impairment (4, 59). It may also provide a basis for future multimodal phenotyping integrating pulmonary function, CV imaging, functional assessment, and biomarkers (6, 59, 63). However, no screening algorithm, risk stratification model, or treatment strategy based specifically on the heart lung fibrotic axis has been prospectively validated. Clinical decisions should therefore continue to follow established pulmonary and CV recommendations until dedicated prospective evidence becomes available. Another unmet need is the development of standardized multimodal screening strategies for CV involvement in IPF. At present, the optimal combination and timing of echocardiographic strain imaging, TAPSE/PASP assessment, CPET, cardiac biomarkers, RHC, and CMR have not been established (4, 6, 12, 36, 37, 59–61, 63). Prospective cohort studies are needed to determine which diagnostic markers provide the greatest incremental value for early detection, risk stratification, and longitudinal monitoring of subclinical myocardial and pulmonary vascular involvement.

The prognostic role of subclinical cardiac abnormalities also remains incompletely defined. While several studies suggest that impaired ventricular strain, abnormal RV–PA coupling, and diffuse myocardial fibrosis may precede overt CV disease, robust evidence linking these findings to mortality, hospitalization, acute exacerbations, or progression of PH in IPF is still insufficient. Longitudinal studies with hard clinical endpoints are therefore essential to determine whether early CV imaging abnormalities should be incorporated into routine risk assessment.

Collectively, these limitations reflect the predominantly observational, cross-sectional, and heterogeneous nature of the current evidence. Many studies include mixed populations of fibrotic ILDs rather than well-characterized cohorts of patients with IPF, while definitions of subclinical cardiac involvement and thresholds for abnormal imaging findings vary substantially. These methodological differences limit direct comparison between studies and prevent the routine incorporation of advanced CV phenotyping into clinical practice.

### 8.3 Therapeutic implications and future research

Important uncertainties also remain regarding the therapeutic implications of integrated cardiopulmonary involvement in IPF. Current treatment remains largely specific to individual organs, with approved antifibrotic therapies targeting the lung and conventional neurohormonal therapy indirectly modifying cardiac remodeling (1, 48, 49, 78, 83, 87, 94, 95). It remains unclear whether antifibrotic therapy targeting the lung modifies the natural history of subclinical cardiac involvement in IPF or whether CV comorbidities influence the efficacy and tolerability of antifibrotic treatment in a clinically meaningful way (2, 85, 86). Although several approaches, including pirfenidone in myocardial fibrosis, TGF-β/ALK5 inhibition, TNIK inhibition, senotherapeutic strategies, and FAP-targeted approaches, provide translational or experimental evidence relevant to fibrotic remodeling, none has prospectively demonstrated modification of a coordinated pulmonary and myocardial fibrotic process in patients with IPF (86, 96–101). Future studies should move beyond conceptual models and investigate whether integrated pulmonary and cardiac phenotyping can identify reproducible cardiopulmonary fibrotic endotypes with distinct prognostic and therapeutic implications. Future research should therefore focus on several priorities. First, large prospective cohorts should integrate pulmonary function, echocardiographic deformation imaging, CMR, biomarkers, and clinical outcomes to define the true prevalence and significance of subclinical CV involvement in IPF. Second, mechanistic studies using transcriptomics, spatial biology, and advanced experimental models should determine whether shared fibrotic signaling networks can identify biologically coherent heart–lung disease phenotypes (40, 53, 57). Third, interventional studies should explore whether therapies targeting common fibrotic pathways can modify both pulmonary and cardiac remodeling, ideally using combined cardiopulmonary endpoints rather than isolated measures of lung function alone (79, 85, 86, 90, 91, 98). Finally, molecular imaging approaches such as FAPI-PET represent a promising strategy for noninvasive assessment of active fibrotic remodeling across organs, although further studies are needed to define their role in integrated cardiopulmonary disease models (65, 66, 101).

Taken together, future progress in this field will depend on shifting from a model in which CV complications are viewed primarily as comorbidities of advanced lung disease toward a more integrated framework that recognizes the possibility of shared fibrotic biology across the heart and lungs. Establishing its diagnostic, prognostic, and therapeutic value remains a key challenge in contemporary IPF research.

## 9 Conclusions

IPF should not be viewed solely as a fibrotic disorder confined to the lungs. CV involvement, including PH, RV dysfunction, CAD, arrhythmias, VTE, HF, and subclinical myocardial abnormalities, represents an important component of the disease burden and may influence symptoms, functional capacity, hospitalization, and survival. An integrated cardiopulmonary assessment should therefore be considered, particularly when exercise limitation or symptom severity appears disproportionate to the extent of pulmonary impairment.

The concept of a heart–lung fibrotic axis provides a useful framework for integrating clinical CV complications, pulmonary vascular remodeling, myocardial dysfunction, and shared profibrotic mechanisms. However, this paradigm remains insufficiently validated for routine clinical application. Future prospective studies integrating advanced CV imaging, biomarkers, and pulmonary phenotyping will be essential to determine whether the proposed heart–lung fibrotic axis has reproducible biological, diagnostic, or prognostic validity and whether shared fibrotic mechanisms can identify clinically meaningful therapeutic targets in IPF.
